# Prevalence of Anemia and Associated Factors Among Pregnant Women Attending a Rural Primary Health Care Facility in Delhi

**DOI:** 10.7759/cureus.113281

**Published:** 2026-07-24

**Authors:** Dhivagar Jegadeesan, Vijaykumar Rajendran, Saranya Rajamanickam

**Affiliations:** 1 Community Medicine, Swamy Vivekanandha Medical College Hospital and Research Institute, Namakkal, IND

**Keywords:** anemia, determinants, iron deficiency, iron intake, pregnancy

## Abstract

Background

Anemia during pregnancy remains a major public health problem in India, particularly in rural areas. This study estimated the prevalence of anemia and identified factors associated with anemia among pregnant women attending a rural primary health care facility in Barwala, Delhi.

Methodology

A facility-based cross-sectional study was conducted among 186 pregnant women attending the antenatal clinic at the Rural Health Training Centre (RHTC), Barwala, Delhi from October 2024 to October 2025. Data on socio-demographic, nutritional, menstrual, obstetric, and antenatal care characteristics were collected using a pretested semi-structured questionnaire. Hemoglobin concentration was measured using the HemoCue method, and anemia was classified according to WHO criteria. Multivariable binary logistic regression analysis was performed to identify factors independently associated with anemia.

Results

The prevalence of anemia was 74.7%, with a mean hemoglobin concentration of 9.77 ± 1.60 g/dL. Educational status, occupation, per-capita income, dietary iron intake, iron-folic acid (IFA) supplementation, menstrual spotting, gravida status, and history of worm infestation were significantly associated with anemia. In multivariable binary logistic regression analysis, low dietary iron intake (adjusted odds ratio (AOR): 7.84), unemployment (AOR: 4.98), history of worm infestation (AOR: 4.82), and illiteracy (AOR: 3.89) were independently associated with higher odds of anemia.

Conclusion

Anemia remains highly prevalent among pregnant women attending a rural primary health care facility in Delhi. Factors independently associated with anemia included low dietary iron intake, unemployment, history of worm infestation, illiteracy, inadequate IFA supplementation, low per-capita income, higher gravida status, and menstrual spotting. Strengthening nutrition counseling, improving IFA compliance, promoting dietary iron intake, and integrating routine deworming into antenatal care services may help reduce the burden of maternal anemia.

## Introduction

Anemia in pregnancy is defined by WHO as a hemoglobin concentration below 11.0 g/dL [1]. It remains a major public health problem worldwide. According to the WHO, the global prevalence of anemia among pregnant women was 35.5% in 2023, with a substantially higher burden in low- and middle-income countries than in high-income countries [2]. It is associated with adverse maternal and fetal outcomes, including preterm birth, low birth weight, increased susceptibility to infections, and maternal mortality [3]. A review by Mangla and Singla reported that the prevalence of anemia exceeded 20% in nearly 80% of countries worldwide, while remaining below 20% in most industrialized nations [4]. Iron deficiency accounts for more than half of all anemia cases and remains the leading nutritional cause of anemia during pregnancy [5].

The South-East Asian region bears a disproportionately high burden of maternal anemia, with a reported prevalence of 48.7% [6]. According to the National Family Health Survey (NFHS)-4, 50.3% of pregnant women in India and 46.1% in Delhi were anemic [7]. The NFHS-5 reported a prevalence of 52.2% among pregnant women in India and 42.2% in Delhi, indicating only a modest decline over time [8]. However, rural-specific estimates for anemia among pregnant women in Delhi are not available.

The burden of anemia across all three trimesters of pregnancy is influenced by multiple sociodemographic and obstetric factors [9]. India belongs to high anemia-prevalent areas where ≥40% of menstruating adult women and adolescent girls are anemic. India ranked 170th among 180 countries for anemia among females during the Global Nutrition Survey, 2016 [10].

Anemia during pregnancy is a multifactorial condition influenced by nutritional, socioeconomic, obstetric, and infectious factors [11]. Balcha et al. reported that low family income, multiparity, short interpregnancy gap, not taking iron and folate, and poor knowledge of anemia are major contributors to anemia among pregnant women [12]. Brooker et al. highlighted the role of parasitic infestations, particularly hookworm infection, in the development of iron deficiency anemia and demonstrated the effectiveness of preventive interventions such as iron supplementation and deworming [13]. Ghosh-Jerath et al. identified poverty, illiteracy, migration, and high parity as important determinants of inadequate antenatal care utilization [14]. Ramesh et al. observed a significantly higher prevalence of anemia among multigravida women than primigravida women [15]. Similarly, Tomar et al. reported that low socioeconomic status, short birth spacing, and inadequate iron-folic acid (IFA) tablet consumption were significantly associated with anemia during pregnancy [16]. These findings indicate that maternal anemia is shaped by a complex interaction of nutritional, socioeconomic, obstetric, and health-service-related factors.

Although several studies have examined anemia among pregnant women in different parts of India, evidence from rural areas of Delhi remains limited. Local data on the prevalence and factors associated with anemia are essential for planning targeted interventions and strengthening antenatal care services. Therefore, the present study was undertaken to determine the prevalence of anemia and identify sociodemographic, nutritional, menstrual, obstetric, and history of worm infestation factors associated with anemia among pregnant women attending the antenatal clinic of the Rural Health Training Centre (RHTC), Barwala, Delhi.

## Materials and methods

Study setting and design

This facility-based cross-sectional study was conducted at the RHTC, Barwala, North-West Delhi, India. The study was carried out over a one-year period from October 2024 to October 2025. Pregnant women attending the antenatal clinic at the RHTC during the study period were enrolled consecutively.

Study participants and eligibility criteria

The study population comprised pregnant women of any trimester attending the antenatal clinic at RHTC, Barwala. Women who provided written informed consent and were available for spot hemoglobin estimation were included in the study. Pregnant women with known hemoglobinopathies or chronic medical conditions were excluded.

Sample size and sampling technique

The sample size was calculated using the prevalence of anemia among pregnant women (86%) reported by Raj et al. [17]. Applying a 95% confidence level (Z=1.96), p=86% (0.8), q=1−p=14% (0.14), absolute precision of 5% d=0.05, and the formula, \[ n=\frac{Z^{2}p(1-p)}{d^{2}} \], by substituting the values, the minimum required sample size was estimated to be 186. 

A non-probability consecutive sampling technique was employed. All eligible pregnant women attending the antenatal clinic during the study period were approached consecutively and recruited until the required sample size was achieved. During the study period, 197 pregnant women were screened for eligibility. Eleven women were excluded, including seven who declined to provide written informed consent, one with a known hemoglobinopathy, and three with chronic medical conditions. The remaining 186 eligible pregnant women were enrolled and included in the final analysis.

Data collection

Data were collected using a pretested semi-structured questionnaire through face-to-face interviews. Information on sociodemographic characteristics, obstetric history, menstrual history, dietary practices, IFA supplementation, and history of worm infestation was obtained.

Dietary Assessment

Dietary intake was assessed using a single 24-hour dietary recall administered through the pretested questionnaire. Participants were asked to recall all foods and beverages consumed during the previous 24 hours. Portion sizes were estimated using standard household measures, including cups, glasses, bowls, spoons, and katoris commonly used by the participants. Daily energy (kcal), protein (g), and iron (mg) intakes were calculated using the Indian Food Composition Tables (IFCT), 2017, published by the Indian Council of Medical Research-National Institute of Nutrition (ICMR-NIN). Nutrient intakes were compared with the ICMR-NIN Recommended Dietary Allowances (RDA) for pregnant women and categorized as adequate or inadequate. The cutoffs used were 2250 kcal/day for energy, 78 g/day for protein, and 35 mg/day for iron.

Assessment of IFA Supplementation

Adequate IFA supplementation was defined as the consumption of one tablet daily containing 100 mg elemental iron and 500 μg folic acid for at least 100 days during pregnancy, in accordance with the Anemia Mukt Bharat (AMB) strategy under the National Health Mission.

Hemoglobin Estimation

Hemoglobin concentration was measured using the HemoCue® Hb 201 System (HemoCue AB, Ängelholm, Sweden). Capillary blood was collected by finger prick under aseptic precautions into a disposable HemoCue microcuvette according to the manufacturer's instructions, and hemoglobin concentration was measured immediately using the portable photometer. The HemoCue Hb 201 system measures hemoglobin using the azide methemoglobin method at wavelengths of 570 nm and 880 nm. The instrument was operated according to the manufacturer's recommendations and checked daily using the manufacturer's control cuvette. All measurements were performed by trained investigators using standardized procedures. A single hemoglobin measurement was obtained for each participant.

Anemia Classification

Anemia was classified according to the WHO criteria for pregnant women as mild (10.0-10.9 g/dL), moderate (7.0-9.9 g/dL), and severe (<7.0 g/dL)

Statistical analysis

Data were entered into Microsoft Excel (Microsoft Corp., Redmond, WA, USA) and analyzed using IBM SPSS Statistics version 25.0 (IBM Corp., Armonk, NY, USA). Continuous variables were summarized using means and standard deviations, whereas categorical variables were expressed as frequencies and percentages. Associations between anemia and categorical variables were assessed using the chi-square test. Variables that were statistically significant in the bivariate analysis and those considered clinically relevant were entered into a multivariable binary logistic regression model to identify factors independently associated with anemia. Adjusted odds ratios (AORs) with 95% confidence intervals (CIs) were reported. Statistical significance was set at p<0.05. No missing data were identified; therefore, all 186 participants were included in the final analysis.

Ethical considerations

The study was approved by the Institutional Ethics Committee of Maulana Azad Medical College, New Delhi (Ref. No. F.No.17/IEC/MAMC/2024/Comm.Med./02). Written informed consent was obtained from all participants before enrollment. The Institutional Ethics Committee-approved protocol included objectives to assess the prevalence of anemia and factors associated with anemia, in addition to evaluating the diagnostic validity of clinical pallor. Therefore, all analyses reported in this manuscript were conducted within the scope of the original ethics approval.

## Results

**Figure 1:**
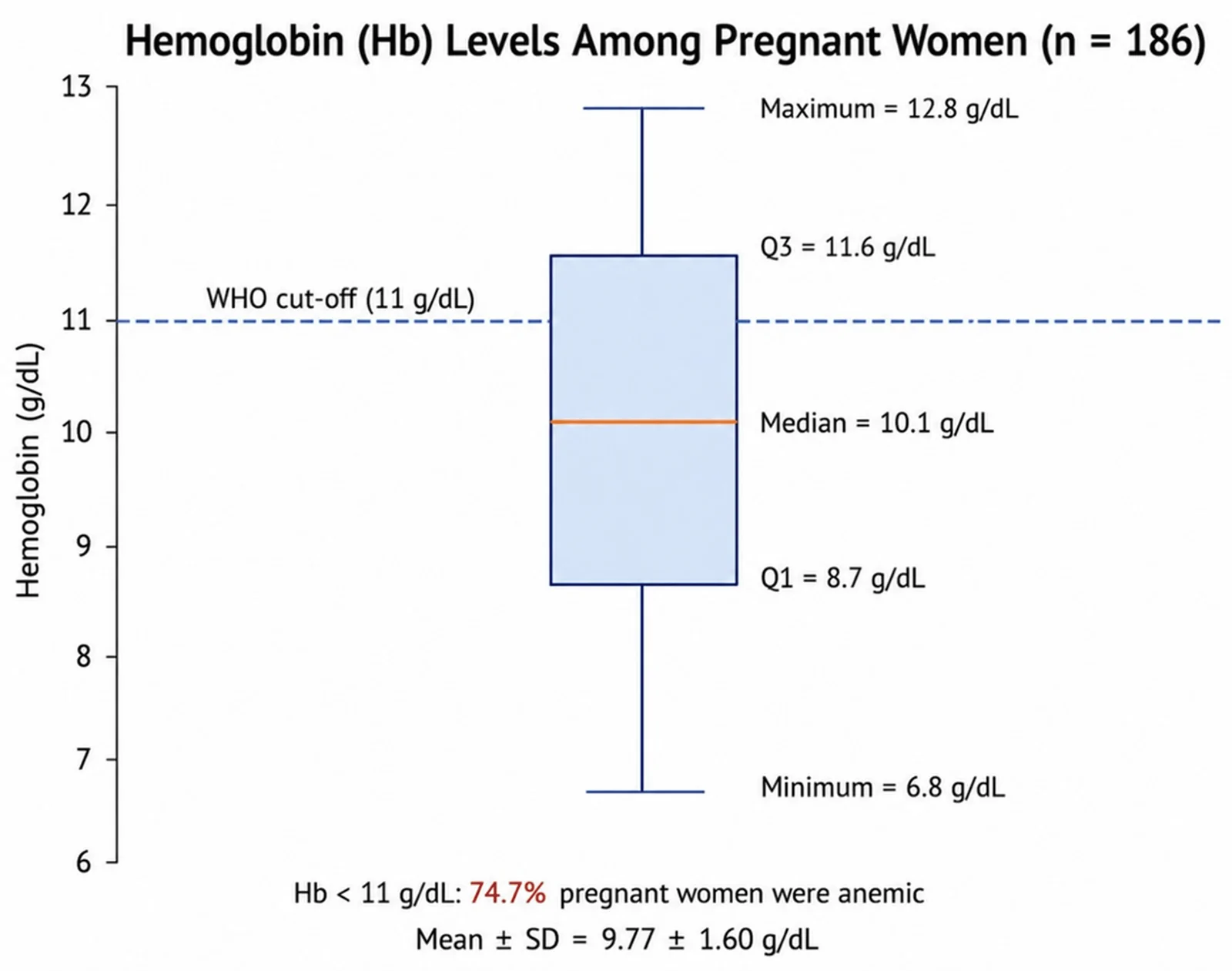
Distribution of hemoglobin levels among pregnant women (n=186)

The median hemoglobin level was 10.1 g/dL (IQR: 8.7-11.6 g/dL), which was below the WHO cut-off of 11 g/dL. The mean hemoglobin concentration was 9.77 ± 1.60 g/dL. Overall, 74.7% of pregnant women were anemic, indicating a substantial burden of anemia in the study population (Figure 1).

**Figure 2:**
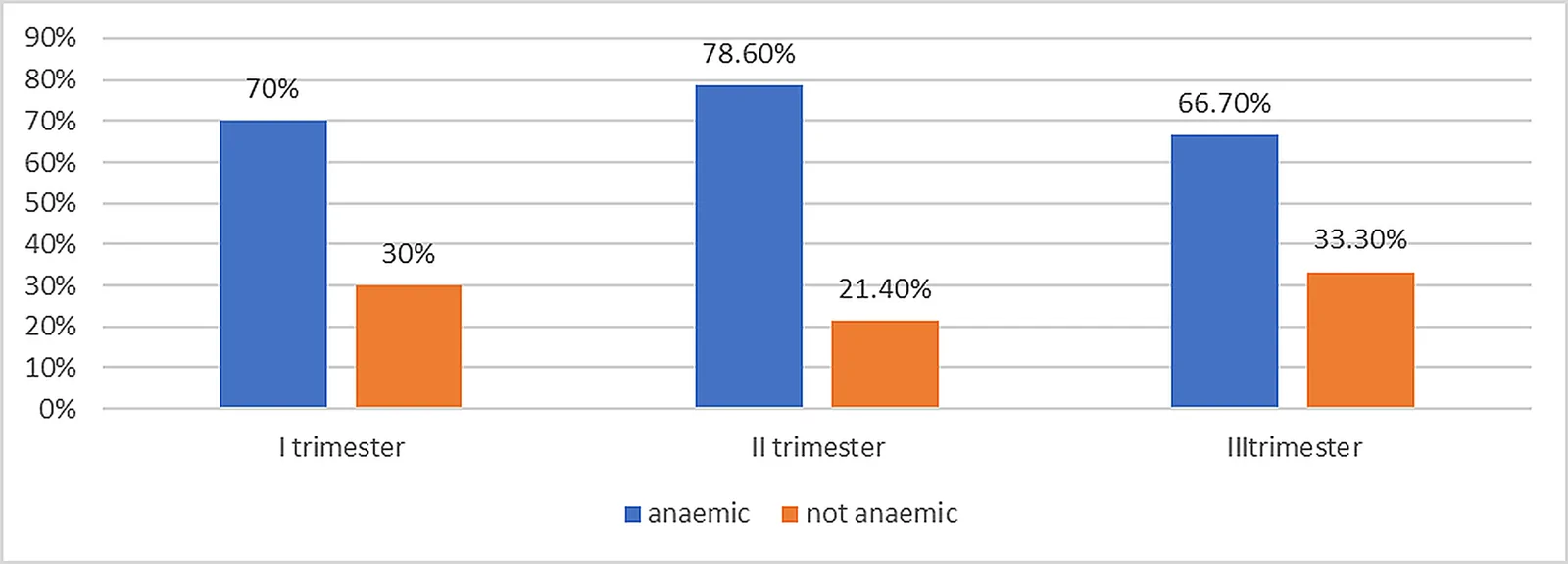
Prevalence of anemia across trimesters of pregnancy (n=186)

The prevalence of anemia was highest during the second trimester (78.6%), followed by the first trimester (70.0%) and third trimester (66.7%). Non-anemic women constituted only 21.4% of second-trimester participants. These findings suggest that anemia remains common throughout pregnancy, particularly during the second trimester (Figure 2).

No statistically significant association was observed between anemia severity and age, caste, religion, or family type (p>0.05). Moderate anemia was the predominant category across most socio-demographic groups. A relatively higher proportion of severe anemia was observed among women from scheduled tribe communities and three-generation families (Table 1).

**Table 1:** Association between socio-demographic characteristics and severity of anemia among pregnant women (n=186) ꭓ2, chi-square test.

Socio-demographic characteristics		Mild anemia, n (%)	Moderate anemia	Severe anemia	Anemia absent	Total	ꭓ^2^	p-value
Age in years	18-24	29 (33.3)	23 (26.4)	9 (10.3)	26 (29.9)	87	4.61	0.59
	25-30	25 (31.3)	29 (36.2)	7 (8.7)	19 (23.8)	80		
	>30	7 (36.8)	8 (42.1)	2 (10.4)	2 (10.4)	19		
Caste	SC	10 (26.3)	15 (39.5)	4 (10.5)	9 (23.7)	38	8.62	0.19
	ST	14 (37.8)	13 (35.1)	6 (16.2)	4 (10.8)	37		
	OBC	37 (33.3)	32 (28.8)	8 (7.2)	34 (30.6)	111		
Religion	Hindu	48 (32.2)	53 (35.6)	13 (8.7)	35 (23.5)	149	4.73	0.57
	Muslim	7 (36.8)	4 (21)	2 (10.5)	6 (31.5)	19		
	Others	6 (33.3)	3 (16.7)	3 (16.7)	6 (33.3)	18		
Type of family	Nuclear	31 (28.7)	37 (34.3)	7 (6.5)	33 (30.5)	108	10.30	0.11
	Joint	11 (33.3)	11 (33.3)	3 (9.1)	8 (24.2)	33		
	Three generation	19 (42.2)	12 (26.7)	8 (17.7)	6 (13.3)	45		

Educational status (p=0.008), occupation (p=0.002), and per-capita income (p=0.002) showed significant associations with anemia severity. Socioeconomic status was classified using the modified BG Prasad scale of 2017, grouping monthly per-capita income into upper (Class I and II), middle (Class III), and lower (Class IV and V) socioeconomic strata. Severe anemia was highest among illiterate women (24.1%) and women from the lower-income group (25.0%), compared to 2.7% among women educated up to high school and above. Anemia was present among 83.8% of unemployed women. Normal hemoglobin levels were highest among women with high school education and above (40.5%) and skilled workers (65.0%) (Table 2).

**Table 2:** Association between educational status, occupation, per-capita income, and severity of anemia among study participants (n=186) ꭓ2, chi-square test. *p<0.05 is statistically significant.

Socio-demographic characteristics	Category	Normal, n (%)	Anemia			Total, n	ꭓ^2^	p-value
			Mild, n (%)	Moderate, n (%)	Severe, n (%)			
Education	Illiterate	3 (10.3)	12 (41.3)	7 (24.1)	7 (24.1)	29	14.10	0.008*
	Primary and middle	29 (24.2)	37 (30.8)	44 (36.6)	10 (8.3)	120		
	High school and above	15 (40.5)	12 (32.4)	9 (24.3)	1 (2.7)	37		
Occupation	Unemployed	18 (16.2)	44 (39.6)	38 (34.2)	11 (9.9)	111	21.54	0.002*
	Unskilled	5 (25)	5 (25)	7 (35)	3 (15)	20		
	Semiskilled	11 (31.4)	8 (22.8)	13 (37.1)	3 (8.5)	35		
	Skilled and above	13 (65)	4 (20)	2 (10)	1 (5)	20		
Per-capita income	Upper	26 (33.3)	34 (43.6)	15 (19.2)	3 (3.8)	78	18.73	0.002*
	Middle	15 (23.4)	20 (31.25)	25 (39)	4 (6.2)	64		
	Lower	6 (13.6)	7 (15.9)	20 (45.4)	11 (25)	44		

Dietary iron intake was significantly associated with anemia severity (p=0.001), whereas calorie intake (p=0.29) and protein intake (p=0.23) were not. Among women consuming <35 mg/day of iron, 83.6% were anemic, including 41.8% with moderate anemia. In contrast, among those consuming ≥35 mg/day, 61.5% had normal hemoglobin levels (Table 3). 

**Table 3:** Association between nutrient intake and severity of anemia among study participants (n=186) ꭓ2, chi-square test. *p<0.05 is statistically significant.

Nutrient intake	Category	Normal, n (%)	Anemia			Total, N	ꭓ^2^	p-value
			Mild, n (%)	Moderate, n (%)	Severe, n (%)			
Calorie intake (kcal/day)	<2250	41 (23.7)	57 (32.9)	58 (33.5)	17 (9.8)	173	3.74	0.29
	>2250	6 (46.1)	4 (30.8)	2 (15.4)	1 (7.7)	13		
Protein intake (g/day)	<78	41 (23.5)	58 (33.3)	58 (33.3)	17 (9.7)	174	4.27	0.23
	>78	6 (50)	3 (25)	2 (16.6)	1 (8.3)	12		
Iron intake (mg/day)	<35	15 (11.2)	48 (35.8)	56 (41.8)	15 (11.2)	134	25.46	0.001^*^
	≥35	32 (61.5)	13 (25)	4 (7.7)	3 (5.7)	52		

Dietary iron intake was significantly associated with anemia severity (p=0.001), whereas calorie intake (p=0.29) and protein intake (p=0.23) were not. Among women consuming <35 mg/day of iron, 83.6% were anemic, including 41.8% with moderate anemia. In contrast, among those consuming ≥35 mg/day, 61.5% had normal hemoglobin levels.

Among first-trimester women, anemia was present in 88.5% of those with inadequate folic acid intake compared with 53.4% among those with adequate intake (p=0.004). In the second trimester, anemia was observed in 92.6% of women with inadequate IFA intake compared with 74.1% among those with adequate intake (p=0.04). The lack of statistical significance in the third trimester (p=0.59) is likely due to low statistical power caused by the small number of participants (n=18) in this subgroup (Table 4).

**Table 4:** Association between folic acid/IFA tablet intake and anemia status during pregnancy among study participants (n=186) ꭓ2, chi-square test; IFA, iron-folic acid. *p<0.05 is statistically significant.

Trimester (supplement)	Intake	Normal, n (%)	Anemic, n (%)	Total, n	ꭓ^2^	p-value
First trimester (folic acid tablet intake)	Adequate	14 (46.6%)	16 (53.4%)	30	8.38	0.004*
	Inadequate	3 (11.5%)	23 (88.5%)	26		
Second trimester (IFA tablet intake)	Adequate	22 (25.9%)	63 (74.1%)	85	4.22	0.04*
	Inadequate	2 (7.4%)	25 (92.6%)	27		
Third trimester (IFA tablet intake)	Adequate	5 (31.2%)	11 (68.8%)	16	0.29	0.59
	Inadequate	1 (50.0%)	1 (50.0%)	2		

Anemia was significantly associated with duration of menstruation (p=0.03), menstrual spotting (p=0.04), gravida status (p=0.002), and history of worm infestation (p=0.001). The prevalence of anemia was 87.0% among women with a history of spotting and 93.3% among those reporting worm infestation. Anemia prevalence increased from 68.4% in primigravida women to 89.3% among gravida 3 women (Table 5).

**Table 5:** Association between menstrual, obstetric, and helminthic factors and anemia status during pregnancy among study participants (n=186) ꭓ2, chi-square test. *p<0.05 is statistically significant.

	Category	Normal, n (%)	Anemic, n (%)	Total, n	ꭓ^2^	p-value
Duration of menstruation	3-7 days	44 (24.8)	133 (75.2)	177	7.02	0.03*
	<3 days	2 (40)	3 (60)	5		
	>7 days	1 (25)	3 (75)	4		
Spotting	Yes	3 (13)	20 (87)	23	4.22	0.04*
	No	44 (27)	119 (73)	163		
Gravida	Primi	36 (31.6)	78 (68.4)	114	12.43	0.002*
	Gravida 2	8 (18.2)	36 (81.8)	44		
	Gravida 3	3 (10.7)	25 (89.3)	28		
History of worms in stools	Yes	3 (6.7)	42 (93.3)	45	11.87	0.001*
	No	44 (31.2)	97 (68.8)	141		

Educational status was dichotomized as illiterate, primary education, and above. Per capita income was categorized as Classes I-III and Classes IV-V according to the Modified BG Prasad socioeconomic classification (Table 6). 

**Table 6:** Multivariable binary logistic regression analysis of factors associated with anemia among pregnant women (n=186) Ref, reference; Wald χ², Wald chi-square statistic. *p<0.05 is statistically significant.

Independent variable	Category	β coefficient	Wald χ²	Adjusted OR (AOR)	95% CI	p-value
Maternal education	Primary and above (ref)	–	–	1.00 (ref)	–	–
	Illiterate	1.36	2.66	3.89	1.76-6.82	0.020*
Maternal occupation	Employed (ref)	–	–	1.00 (ref)	–	–
	Unemployed	1.61	5.57	4.98	1.31-7.91	0.019*
Per-capita income	Class I-III (ref)	–	–	1.00 (ref)	–	–
	Class IV-V	0.46	0.51	1.58	1.05-3.59	0.040*
Dietary iron intake	≥35 mg/day (ref)	–	–	1.00 (ref)	–	–
	<35 mg/day	2.06	8.56	7.84	4.91-12.14	<0.001*
IFA/folic acid intake	Adequate (ref)	–	–	1.00 (ref)	–	–
	Inadequate	1.09	3.91	2.98	1.01-4.79	0.038*
History of worms in stools	No (ref)	–	–	1.00 (ref)	–	–
	Yes	1.57	5.15	4.82	1.24-8.67	0.023*
Obstetric profile (gravida)	Primigravida/gravida 2 (ref)	–	–	1.00 (ref)	–	–
	Gravida 3	0.85	1.28	2.33	1.54-4.12	0.030*
Menstrual spotting history	No (ref)	–	–	1.00 (ref)	–	–
	Yes	0.42	0.72	1.52	1.11-3.41	0.040*

**Figure 3:**
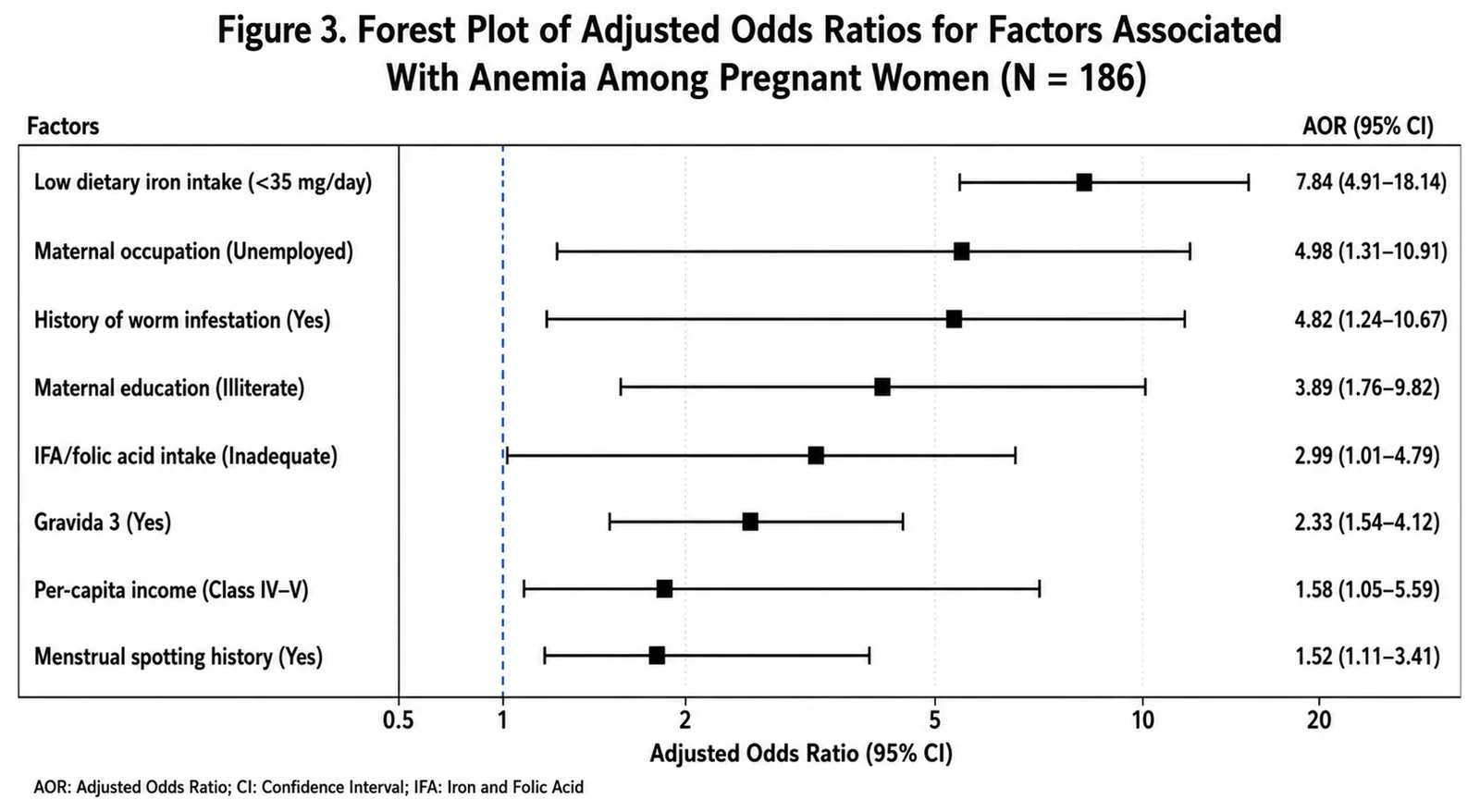
Forest plot of adjusted odds ratios for factors associated with anemia among pregnant women (n=186)

Low dietary iron intake (<35 mg/day) was the factor most strongly associated with anemia (AOR: 7.84) (Table 6), followed by maternal unemployment (AOR: 4.98), history of worm infestation (AOR: 4.82), and illiteracy (AOR: 3.89). Lower socioeconomic status, inadequate IFA supplementation, gravida ≥3, and menstrual spotting were also independently associated with increased odds of anemia (Figure 3).

## Discussion

In this study, 74.7% of pregnant women were anemic, with a mean hemoglobin level of 9.77 ± 1.60 g/dL, which is well below the WHO cut-off of 11 g/dL for defining anemia in pregnancy [1]. The median hemoglobin concentration was 10.1 g/dL (IQR: 8.7-11.6 g/dL), indicating a substantial burden of anemia among the study population. The prevalence observed in the present study was considerably higher than the national estimates reported by the NFHS-4 (50.3%) and NFHS-5 (52.2%) among pregnant women in India [7,17] but comparable with Mangla and Singla (75.4%) [4]. Tomar et al. (67.0%) [16] and Raj and Mangasuli (85.4%) [17] suggest that anemia continues to be a major public health problem among pregnant women in India despite ongoing supplementation programs.

Anemia was most common in the second trimester (78.6%), followed by the first (70.0%) and third (66.7%) trimesters. This finding is consistent with known pregnancy physiology where plasma volume increases by 40%-60%, while red blood cell mass rises by only 20%-50%. This causes dilutional anemia, especially in the second trimester [18]. The higher prevalence of anemia in the second trimester (78.6%) is consistent with physiological hemodilution during pregnancy and increasing iron requirements for maternal and fetal growth [12,18]. Similar trimester-wise patterns have been reported in previous Indian studies [4,16]. The increased anemia rates in the second trimester reflect the need for targeted screening and interventions at all antenatal visits during this critical time.

No significance was found between anemia severity and age, caste, religion, or family type in our study (p>0.05). However, educational status (p=0.008), occupation (p=0.002), and per-capita income (p=0.002) were significantly associated with anemia severity. In this study, severe anemia was most common among illiterate women (24.1%) and those in the lower-income group (25.0%). In contrast, only 2.7% of women with high school education or higher had severe anemia, and the same is reflected in NFHS-5 data [8]. In our study, women without formal education had nearly four times higher odds of severe anemia (AOR: 3.89, 95% CI: 1.76-6.82) than educated women. Similar associations between low educational status and anemia have been reported by Tomar et al. [16] and Ghosh-Jerath et al. [14]. Education helps women get timely antenatal care, know their dietary needs, and take iron supplements. Unemployed pregnant women had significantly higher odds of anemia than employed women (AOR: 4.98, 95% CI: 1.31-10.91). Lower-income women struggle to access iron-rich foods, antenatal services, and supplements. These socioeconomic disparities require targeted health programs to reduce anemia effectively.

Low dietary iron intake (<35 mg/day) was the factor most strongly and independently associated with anemia in the multivariable logistic regression analysis (AOR: 7.84). Among women consuming less than 35 mg/day of dietary iron, 83.6% were anemic, including 41.8% with moderate anemia. Iron deficiency is the leading cause of anemia in pregnancy, accounting for about 50% of all cases worldwide. Adherence to IFA supplementation also significantly influenced anemia rates. Anemia affected 88.5% of women with inadequate folic acid intake in the first trimester (p=0.004) and 92.6% of those lacking IFA intakes in the second trimester (p=0.04). This aligns with findings from North India, where consistent IFA use was strongly linked to lower anemia rates (OR=0.26, 95% CI: 0.12-0.55) [19]. However, uptake remains low due to supply chain gaps and demand-side barriers. These findings denotes the importance of strong supply chains, Accredited Social Health Activist-led counseling, and early antenatal care registration. This ensures continuous supplementation starting from the first trimester.

A history of worm infestation was also independently associated with anemia (AOR: 4.82), with 93.3% being anemic among them. Soil-transmitted helminths, such as hookworms, cause chronic blood loss. This impairs iron absorption and can lead to anemia in women of reproductive age. A Cochrane review found that intestinal helminthiasis leads to blood loss and less nutrients for blood production. This causes iron deficiency anemia, impacting 44 million pregnancies globally each year [20]. Despite this, deworming treatments are underused in antenatal care program in India. Routine deworming with albendazole after the first trimester should be integrated into antenatal services, especially in endemic areas, to tackle this preventable cause of maternal anemia.

Higher gravida status was independently associated with increased odds of anemia. The prevalence of anemia increased from 68.4% among primigravid women to 89.3% among women with three or more pregnancies (AOR: 2.33). This finding supports the concept that repeated pregnancies progressively deplete maternal iron stores when adequate replenishment does not occur between pregnancies. Multiparous women have been reported to have lower serum ferritin concentrations than nulliparous women, increasing their risk of anemia during pregnancy and at delivery. Similarly, Munro et al. reported that multiparity was associated with an increased risk of anemia, whereas primigravid women were 61% less likely to be anemic than multigravida women [21]. These findings underscore the importance of preconception nutritional optimization, adequate iron supplementation, and appropriate interpregnancy intervals to restore maternal iron stores and reduce the risk of anemia in subsequent pregnancies.

A history of menstrual spotting was also independently associated with anemia (AOR: 1.52), with 87.0% of these women being anemic, consistent with the findings of Sinha et al. [18]. Chronic blood loss resulting from abnormal uterine bleeding before pregnancy may contribute to depleted iron stores and increase the risk of anemia during pregnancy. Menstrual abnormalities are often overlooked as a contributing factor to maternal anemia. These findings highlight the importance of screening for menstrual disorders during the preconception period, providing timely gynecological evaluation and treatment, and strengthening the AMB program to improve iron status before pregnancy.

Study strengths

This study provides recent evidence on the prevalence and determinants of anemia among pregnant women in a rural primary care setting. Hemoglobin estimation was performed using the validated HemoCue point-of-care device, ensuring reliable assessment, and a comprehensive evaluation of sociodemographic, obstetric, dietary, and supplementation-related factors was undertaken. The study provides important evidence on the burden and determinants of anemia among pregnant women in a rural primary care setting, supporting the need for strengthened screening and targeted interventions.

Study limitations

This study was limited by its facility-based cross-sectional design, and consecutive recruitment from a single rural antenatal clinic may have introduced selection bias and limited the generalizability of the findings. The cross-sectional design also precludes establishing temporal relationships or causal inferences. Information on dietary intake, IFA supplementation, and history of worm infestation was self-reported and may be subject to recall bias. Although hemoglobin estimation using the HemoCue system is a validated point-of-care method, confirmatory laboratory investigations were not performed. 

## Conclusions

Anemia was highly prevalent among pregnant women attending a rural primary health care facility in Barwala, Delhi. Low dietary iron intake, maternal unemployment, a history of worm infestation, illiteracy, higher gravida status (≥3), inadequate IFA supplementation, and a history of menstrual spotting were independently associated with anemia. Strengthening nutrition counseling, improving adherence to IFA supplementation, promoting routine deworming, encouraging early antenatal care registration, and addressing socioeconomic vulnerabilities through comprehensive antenatal care services may help reduce the burden of maternal anemia.

## Appendices

Questionnaire

(A) Socio-Demographic Profile

**Table 7:** Identification and sociodemographic information

Patient unique ID	
Date of interview	_____/_____/_______
Name	
Age (in completed years)	
Duration of residence	
Native residence	Same/other
Religion	Hindu/Muslim/Sikh/Christian/other
Caste	SC/ST/OBC/other
Type of family	Nuclear/joint/three-generation
Number of family members	
Number of children	
Combined family income	
Per capita income	

**Table 8:** Family member details

Relation	Age (years)	Education	Occupation	Income	Other information
Self					
Husband					
Mother-in-law					
Father-in-law					

(B) History

**Table 9:** 24-hour dietary history

S. no	Time of meal	Food items	Quantity	
1	Early morning			
2	Breakfast			
3	Mid-morning			
4	Lunch			
5	Evening snacks			
6	Dinner			
7	Bed time			
8	Others			

**Table 10:** Food habits For both plant- and animal-derived sources, the frequencies will be numbered as: 0-Never, 1-Occasionally, 2-Three to four times per week, 3-Daily

Plant-derived sources		Animal-derived sources	
Food item	Frequency	Food item	Frequency
Fruits		Chicken	
Green leafy vegetables		Mutton	
Other vegetables		Fish	
Dry fruits		Egg	
Jaggery		Milk and dairy products	
Others		Others	

**Table 11:** Dietary intake assessment

Dietary intake	As per need/not as per need (deficiency)
Protein intake	
Calorie intake	
Iron intake	

**Table 12:** Menstrual history

Question	Response
Age at menarche	__________ years
Duration of menstrual cycle	__________ days
Duration of periods per cycle	__________ days
Does bleeding or spotting occur between periods?	Yes/no
Is pain associated with periods?	Yes/no/occasionally
If yes, what is the duration of pain in relation to periods?	________ days to ________ days before/after onset of periods
Type of napkins used	Sanitary napkins from government supply/sanitary napkins purchased from shop/cotton/used cloth
Maximum number of napkins changed per day during the menstrual period	__________
History of blood clots, if any	Yes/no

**Table 13:** Past obstetric history ^*^Birth outcome: 1 = Live birth; 2 = Stillbirth; 3 = Early neonatal death; 4 = Spontaneous abortion; 5 = Medical termination of pregnancy. ^**^Number of babies born: 1 = Singleton; 2 = Twins; 3 = Triplets; 4 = Multiple. ^***^Type of delivery: 1 = Normal vaginal delivery; 2 = Vaginal delivery with forceps; 3 = Vaginal delivery with vacuum; 4 = Caesarean delivery; 5 = Other modes of delivery.

Pregnancies till now (gravida)	1st	2nd	3rd	4th	5th
Birth outcome^*^					
Number of babies born^**^					
Date of delivery or abortion (--/--/----)					
Day of death of baby (if any)					
Place of delivery/abortion					
Duration of pregnancy					
Hours of labor					
Type of delivery^***^					
Any complications of pregnancy					
Any complications infant had					
Gender of baby	M/F	M/F	M/F	M/F	M/F
Birth weight of baby (grams)					
Present health of the child					
Birth spacing ≥3 years: yes/no					

**Table 14:** Present obstetric history

Present obstetric history	Response
(i) GPLA	
(ii) LMP	
(iii) EDD	
(iv) Period of gestation	
(v) Do you know what is ANC?	Yes/no
(vi) Is this your first ANC visit?	Yes/no
(vii) If "no" to question (vi), number of ANC visits taken excluding this	
(viii) Do you have ANC card?	Yes/no
(ix) Date of registration in ANC	
(x) From where you are getting ANC check-ups	
(xi) Number of TT taken	
(xii) How many tablets of IFA taken/out of expected	__________ / __________
(xiii) What are all the investigations you have already done	

**Table 15:** History of conditions associated with anemia

History of conditions associated with anemia	Yes/no	Past or present history	Treatment taken
(i) History of worms in stools	Yes/no		
(ii) History of any form of blood loss from any natural orifices	Yes/no		
(iii) History of any form of blood loss due to injury	Yes/no		
(iv) History of pica	Yes/no		
(v) History of any disease in the last three months	Yes/no		
(vi) History of any blood loss during present or past pregnancy	Yes/no		
(vii) Other symptoms with duration, if any	Yes/no		
(viii) Any other cause(s) of anemia present	Yes/no		

(C) Symptoms

**Table 16:** Anemia-related symptoms at present

Symptoms	Yes/no	Duration
i) Easy fatigability	Yes/no	
ii) Palpitation	Yes/no	
iii) Dizziness	Yes/no	
iv) Shortness of breath	Yes/no	
v) Chest pain	Yes/no	
vi) Trouble concentrating in work	Yes/no	

(D) Examination

**Table 17:** Examintaion

Section	Parameter	Response
1. Anthropometric measurements	Height	cm
	Weight	kg
2. General examination	Bilateral pedal edema	Yes/no
	Pallor	Absent/mild-to-moderate anemia/severe anemia
	Jaundice	Yes/no
	Clubbing	Yes/no
	Pulse rate per minute	
	Respiratory rate per minute	
	Temperature	
	Blood pressure	_____ / _____ mmHg
3. Examination of pallor at different sites	Palpebral conjunctiva	Present/absent
	Nail bed	Present/absent/could not be assessed
	Palm	Present/absent/could not be assessed
	Palmar crease	Present/absent/could not be assessed
4. Systemic examination	Cardiovascular system	
	Respiratory system	
5. Obstetric examination	(i) Inspection	
	Previous cesarean scars	Present/absent/not applicable
	Striae gravidarum	Present/absent/not applicable
	Linea nigra	Present/absent/not applicable
	Visible fetal movements	Present/absent/not applicable
	(ii) Palpation	
	Fundal height	__________ corresponds to __________ weeks of gestation/not applicable
	Fetal movements	Present/absent/not applicable
	Fetal presentation	Cephalic/breech/transverse lie/others/not applicable
	(iii) Auscultation	
	Fetal heart rate	__________ per minute/not applicable

 *(E) Investigation*

**Table 18:** Investigations done/reports

Investigations done/reports	Response
(i) Spot hemoglobin level by HemoCue method	________ gm%
(ii) Grading of anemia according to WHO criteria	Mild anemia/moderate anemia/severe anemia
